# Designing and Implementing an ANFIS Based Medical Decision Support System to Predict Chronic Kidney Disease Progression

**DOI:** 10.3389/fphys.2018.01753

**Published:** 2018-12-06

**Authors:** Ali Yadollahpour, Jamshid Nourozi, Seyed Ahmad Mirbagheri, Eric Simancas-Acevedo, Francisco R. Trejo-Macotela

**Affiliations:** ^1^Department of Medical Physics, School of Medicine, Ahvaz Jundishapur University of Medical Sciences, Ahvaz, Iran; ^2^Department of Environmental and Energy, Science and Research Branch, Islamic Azad University, Tehran, Iran; ^3^Department of Civil and Environmental Engineering, K. N. Toosi University of Technology, Tehran, Iran; ^4^Telematics Engineering Department, Polytechnic University of Pachuca, Zempoala, Mexico; ^5^Graduate and Research Department, Polytechnic University of Pachuca, Zempoala, Mexico

**Keywords:** chronic kidney disease, adaptive neuro fuzzy inference system, medical decision support system, renal failure progression, prediction

## Abstract

**Background and objective:** Chronic kidney disease (CKD) has a covert nature in its early stages that could postpone its diagnosis. Early diagnosis can reduce or prevent the progression of renal damage. The present study introduces an expert medical decision support system (MDSS) based on adaptive neuro-fuzzy inference system (ANFIS) to predict the timeframe of renal failure.

**Methods:** The core system of the MDSS is a Takagi-Sugeno type ANFIS model that predicts the glomerular filtration rate (GFR) values as the biological marker of the renal failure. The model uses 10-year clinical records of newly diagnosed CKD patients and considers the threshold value of 15 cc/kg/min/1.73 m^2^ of GFR as the marker of renal failure. Following the evaluation of 10 variables, the ANFIS model uses the weight, diastolic blood pressure, and diabetes mellitus as underlying disease, and current GFR_(*t*)_ as the inputs of the predicting model to predict the GFR values at future intervals. Then, a user-friendly graphical user interface of the model was built in MATLAB, in which the user can enter the physiological parameters obtained from patient recordings to determine the renal failure time as the output.

**Results:** Assessing the performance of the MDSS against the real data of male and female CKD patients showed that this decision support model could accurately estimate GFR variations in all sequential periods of 6, 12, and 18 months, with a normalized mean absolute error lower than 5%. Despite the high uncertainties of the human body and the dynamic nature of CKD progression, our model can accurately predict the GFR variations at long future periods.

**Conclusions:** The MDSS GUI could be useful in medical centers and used by experts to predict renal failure progression and, through taking effective actions, CKD can be prevented or effectively delayed.

## Introduction

Early and differential diagnosis or prediction of disease progression and improving diagnosis reliability are today becoming prerequisite needs in medicine and health systems (Anagnostou et al., 2003; Al-Shayea et al., 2013). In this regard, developing expert and intelligent systems based on machine learning approaches for efficient diagnosis, prediction, and effective management of diseases has drawn considerable research attention among physicians and researchers (Anagnostou et al., 2003; Ohlsson, 2004; Ubeyli and Güler, 2005; Parthiban and Subramanian, 2008; Pandey and Mishra, 2009; Lee and Wang, 2011; Al-Shayea et al., 2013). Decision support systems (DSSs) taking advantages of recent advances in software and computational knowledge in medicine can reduce or even prevent the adverse effects of diseases (Hunt et al., 1998; Montgomery et al., 2000; Garg et al., 2005; Pandey and Mishra, 2009; Lee and Wang, 2011). Medical decision support systems (MDSSs) are intelligent and expert systems used in medicine to help medical experts make appropriate decisions in different fields (Smith et al., 2003). Physicians and health experts could use these systems to eliminate the biases associated with humans, such as tiredness, and environmental interfering factors and to make knowledge-based decisions (Ohlsson, 2004; Garg et al., 2005). Artificial intelligence has gained considerable research interest and has been utilized in different domains of modern medicine such as alarm producing, reminders, and approving diagnostic decisions. In medicine, there is an integrated relationship between data and knowledge, in which knowledge of detection, diagnosis, interpretation, and treatment of a disease is influenced by the data of that disease (Miller, 1994). The level of this relation varies depending on the disease type and interfering and biasing factors. In this regard, intelligent computing models as a category of artificial intelligence prefer to work with data rather than knowledge (Pandey and Mishra, 2009). The artificial intelligence-based MDSSs compared with other MDSSs are more robust and rigorous systems (Ohlsson, 2004; Hayward et al., 2010). The MDSSs can reduce uncertainties and processing time, and produce the knowledge extracted from raw data to be used by medical experts. Computer-based MDSSs can be implemented in web-based frameworks with full time online access in healthcare systems (Hunt et al., 1998; Ohlsson, 2004; Garg et al., 2005). The adaptive neuro-fuzzy inference system (ANFIS) integrates neural networks and fuzzy logic principles into a single framework with learning capability to approximate non-linear functions and works as a universal estimator (Jang, 1992, 1993). ANFIS is a type of neural network, based on the Takagi–Sugeno fuzzy inference system, developed in the early 1990s. The learning networks in this model are based on mathematical computations capable of solving complex problems. ANFIS-based predicting models resembling human brain functions can accurately predict diseases (Jang and Sun, 1997; Ubeyli and Güler, 2005). This system has been used for predicting the onset, classification or differential diagnosis of different disorders including heart failure, cognitive disorders, Alzheimer disease, ischemia, multiple sclerosis, etc. (Brier et al., 2003; Lauer et al., 2005; Ubeyli and Güler, 2005; Ercelebi and Subasi, 2006; Krug et al., 2008; Emam et al., 2010; Al-Kasasbeh et al., 2011; Yadollahpour and Jalilifar, 2014; Norouzi et al., 2016; Zhao et al., 2017). Clinical decisions based on fuzzy logic models are cost effective and beneficial in improving healthcare systems. One of the significant features of fuzzy logic approaches is removing uncertainties of dynamic systems. Uncertainties can be represented and controlled effectively (Parthiban and Subramanian, 2008). Fuzzy logic models were used in predicting appendicitis with high accuracy (Pandey and Mishra, 2009; Al-Shayea et al., 2013). Different expert systems have been used in medicine (Buchanan and Shortliffe, 1984; Ohlsson, 2004; Lee and Wang, 2011; Ma, 2012). MYCIN is one of the early clinical decision systems developed at Stanford University in the early 1970s. This DSS was an expert system based on artificial intelligence to identify bacteria causing severe infections in order to recommend specific antibiotics for patients (Buchanan and Shortliffe, 1984). This DSS used the results of physical and laboratory tests of subjects to diagnose blood infections. Chronic kidney disease (CKD) is a global health problem with 8 to 16 percent worldwide prevalence (Nugent et al., 2011). The prevalence of the disease has dramatically increased worldwide (Meguid El Nahas and Bello, 2005). Variations in population censuses, different diagnoses in different racial populations, a lack of diagnosis in early stages and risk factors contribute to the rapid increase of the disease (Coresh et al., 2002; Bello et al., 2005; El Nahas, 2005). Annual costs of CKD patients are high, and so the disease imposes high costs on each country. Annual costs of CKD patients in the USA exceeded 39.46 million US$ for 2008, equaling 23 percent of healthcare expenditures (Coresh et al., 2002). Despite the high medical costs and advances in dialysis treatments for CKD patients, mortality and morbidity rates of the disease are still high and the patients have a low quality of life (Pickle et al., 1996).

Reviewing the recent literature in MDSSs showed that fuzzy intelligent systems, especially neuro-fuzzy inference systems, have been widely used in predicting the state and progression of various diseases (Ubeyli and Güler, 2005; Parthiban and Subramanian, 2008; Lee and Wang, 2011). Findings of these studies showed that utilizing these expert systems in combination with the knowledge and diagnosis of clinical specialists can significantly reduce diagnostic errors. Such systems yield more accuracy compared with machine learning techniques.

Early diagnosis of CKD disease is a necessary step to reduce or even prevent the progression of renal damage. Because of the covert nature of the disease in early stages, the uncertainties governing the disease's status, and progression resulting from dynamic features of the human body, we need a robust model to accurately predict the disease's progression. To our knowledge there has not been any study using fuzzy intelligent systems to build a DSS to predict the time of renal failure. In this study we propose a MDSS based on the ANFIS system to predict renal failure progression. We first designed three models to predict the worsening time frame of CKD, including linear regression, multilayer perceptron neural network, and ANFIS models to assess their accuracy (data not presented). Among the three predicting models, ANFIS showed the greatest accuracy (more than 95%) in predicting GFR (Norouzi et al., 2016). Therefore, in this study, we used the ANFIS as the core computing model to build an MDSS for predicting renal failure in CKD patients with a user-friendly interface.

In computerized MDSSs different techniques and algorithms can be used, including rule based reasoning, case based reasoning and machine learning techniques (Pandey and Mishra, 2009). Among these methods, various machine learning-based methods have been used in medicine and healthcare, such as decision trees, artificial intelligence network support vector machines, and Bayesian networks. However, fuzzy intelligent techniques have not yet been used to predict worsening renal function. Furthermore, no MDSS is available to monitor kidney disease progression or to predict the appropriate time for renal replacement therapy (RRT) in CKD patients. ANFIS systems have significant potential for predicting systems high in uncertainty and with a highly dynamic nature, such as CKD progression in the human body as the modeled environment (Parthiban and Subramanian, 2008; Ghumbre and Ghatol, 2010).

## Experimental Section

### Patients and Data Collection

All the data of the present study were comprised of the clinical records of a cohort study of newly diagnosed CKD patients who were serially admitted to the Clinic of Nephrology, Imam Khomeini Hospital (Tehran, Iran) during October 2002-October 2011. All the procedures of the present study were approved by the ethics committee of the Tehran University of Medical Sciences, which coincide completely with the Declaration of Helsinki Ethical Principles for Medical Research Involving Human Subjects (General Assembly of the World Medical Association, 2014). Written consent was obtained from all patients who participated in the study. The inclusion criteria for CKD were small-sized kidneys in ultrasound images or GFR <60 cc/kg/min/1.73 m^2^ for more than 3 months. All the patients whose records were used for the analysis had visited the clinic for at least 6 months. A total of 465 CKD patients participated in the study. They were divided into two groups according to their adherence patterns to the follow-up schedule in the clinic. The test group consisted of 389 patients who continuously (at least every 6 months) were visited in the clinic. The control group consisted of 76 patients who did not regularly follow their visit schedule in the clinic. The patients whose visits postponed for at least 1 year were categorized as control group.

**Figure 1:**
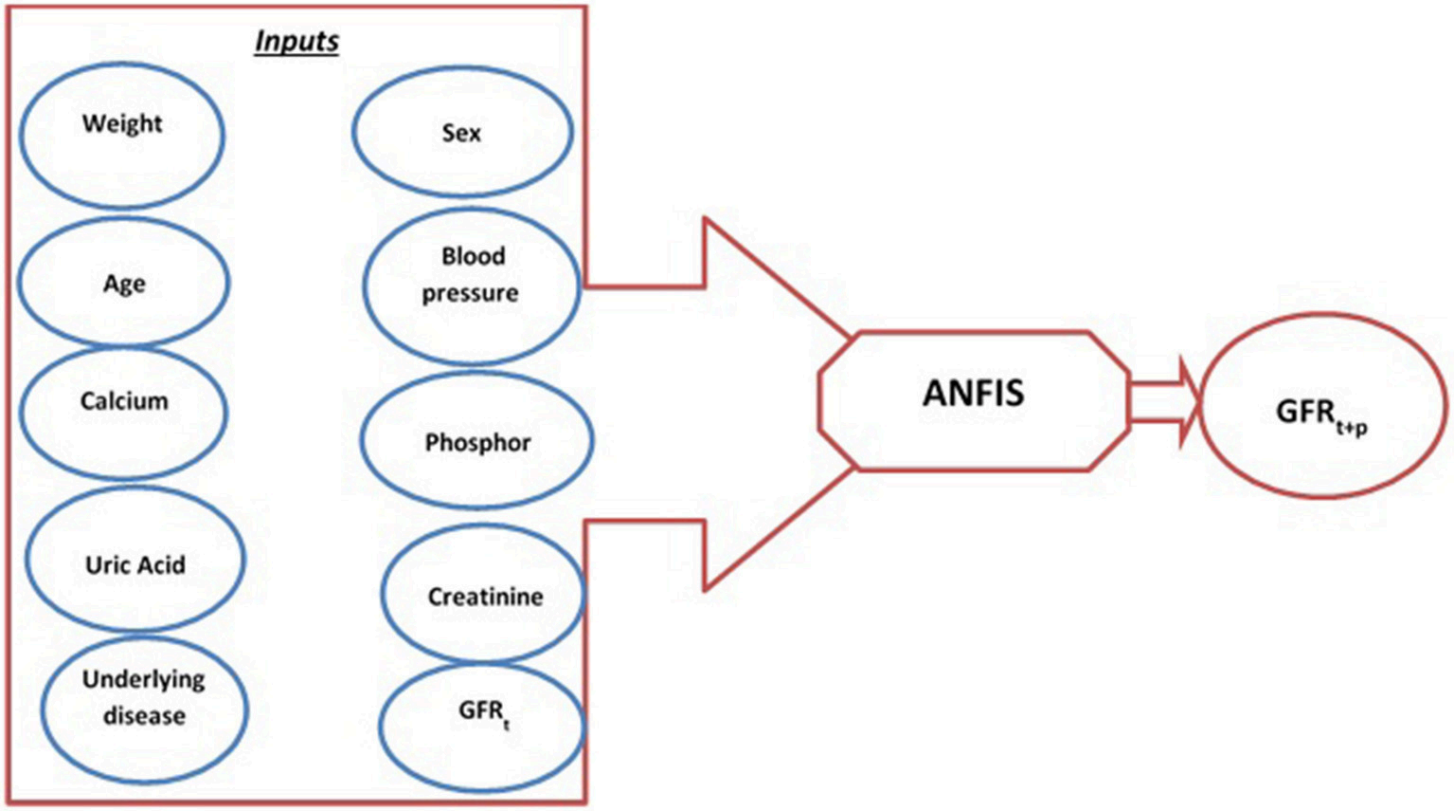
Schematic diagram of predicting model and input variables.

At each visit, a set of clinical and physiological parameters was recorded and monitored for each patient. The variables included the patient's weight, diastolic and systolic blood pressure, serum creatinine level, fasting plasma glucose, lipid profile, calcium, phosphorus, hemoglobin, uric acid, and GFR. Then, the patients were administered suitable treatments for blood pressure, bone mineral metabolism indices, and hemoglobin control. The GFR was estimated using the Modification of Diet in Renal Disease (MDRD) equation. The end point for the patient's follow-up was a GFR value <15 cc/kg/min/1.73 m^2^ or start of RRT or patient death. The MDRD is a formula for estimating GFR based on creatinine and patient characteristics (Levey et al., 1999). This equation is used only for CKD patients, so for acute kidney failure it may result in an inaccurate estimation. All quantitative variables were considered as continuous to have a better training of the model. The recorded clinical and physiological variables along with the demographic data were used to create a dataset. We initially selected 10 variables that were expected to influence the CKD status and progression based on the previous studies and the viewpoints of nephrology specialists (Figure 1). These variables were then used as the input of the predicting models to calculate GFR values at future intervals. The variables included age, sex, weight, underlying diseases, diastolic blood pressure, creatinine, calcium, phosphorus, uric acid, and GFR. In next step, Pearson's correlation coefficient test was used to determine the variables that significantly influence the GFR values. The four variables weight, diastolic blood pressure, diabetes mellitus as underlying disease, and current GFR showed significant correlation with GFR values and were selected as the inputs of the GFR predicting models.

To develop an MDSS for predicting renal failure we first should develop a predicting model to forecast the renal failure time and the time frame where kidney disease worsens. According to the clinical measurements (independent physiological parameters) and clinical outcomes, when GFR reaches <15cc/kg/min/1.73 m^2^, RRT including dialysis or transplant is necessary for the patient's survival (Gaspari et al., 2004). Regarding the previous studies and clinical data, GFR is the only reliable parameter of renal function and progression of CKD (Gaspari et al., 2004). Therefore, to predict renal failure, GFR values should be predicted over time. Predicting the variations of GFR values, we can predict the time at which the GFR reaches the threshold value of 15 cc/kg/min/1.73 m^2^ indicating the time for RRT. The real data recorded during a 10-year period were recorded at 6-month intervals. Therefore, the GFR values were predicted for three sequential 6-month intervals at 6-, 12-, and 18-month intervals. The GFR_(t+p)_ with *p* = 1,2,3 represents the GFR values at 6-, 12-, and 18-month intervals.

We used three predicting models, including improved linear regression, ANFIS, and multilayer perceptron neural networks, to predict the GFR in future time intervals (data not presented). Among the three predicting models, ANFIS was capable of accurately predicting GFR with more than 95% precision (Norouzi et al., 2016). Therefore, ANFIS was used as a core predicting model in building the MDSS.

### Model Description

**Figure 2:**
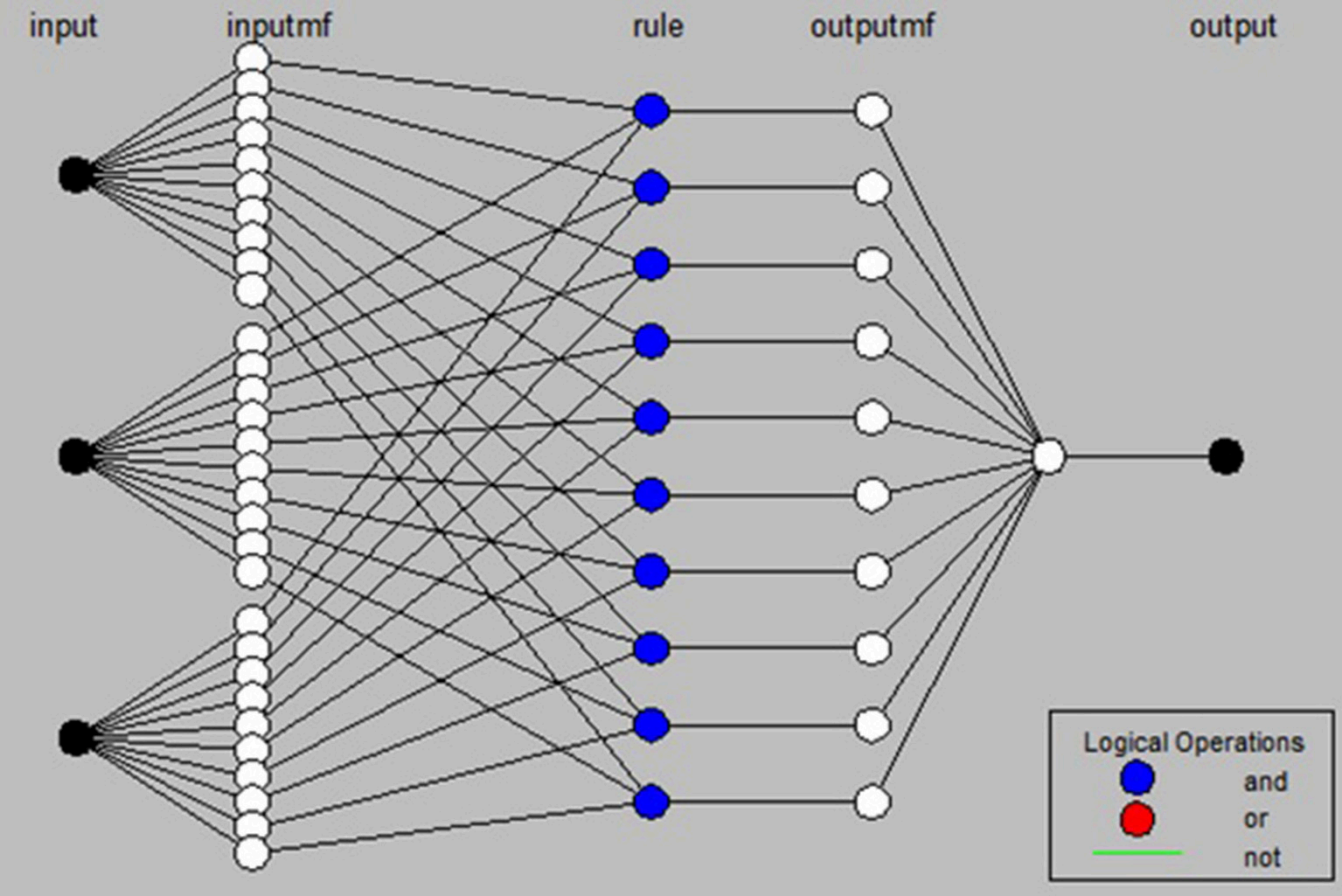
Structure of the ANFIS model used in the present study.

The ANFIS model used in the present study (Figure 2) is based on the model proposed by Jang (Jang, 1992), which is a learner network equivalent to the Takagi-Sugeno fuzzy inference system. Learning in this network is a continuous update of the network parameters. Factors of layer I and layer IV are of the learner type. Factors of the first layer determine membership functions. Factors of layer IV determine the first-order estimated function. The ANFIS training algorithm is a hybrid algorithm, which uses the ordinary least squares algorithm to update coefficients of output functions (fi), while the error back propagation algorithm is used to update fundamental factors of the system (Jang, 1992; Jang and Sun, 1997).

The results of our predicting models support the previous findings, which showed a higher predicting efficacy from ANFIS compared to the linear regression and multilayer perceptron neural network (Norouzi et al., 2016). The ANFIS model could predict the GFR values for future 6-month intervals with normalized mean square error lower than 5%. Therefore, the ANFIS model was used to build the MDSS system. The detailed features of the predicting model can be found in Norouzi et al. (2016). In brief, the ANFIS model works as below: Data of 465 CKD patients were divided into training and test datasets. Training data were used to optimize the weights and other parameters in the model. The test data were used to evaluate the quality of estimates and forecasts. In all further processing and modeling, the test dataset was not used for training models. The test data were randomly selected so that all data had an equal chance to participate in the selection process. The test dataset is usually selected from 30 to 40% of the available data. In this study, 30 and 70% of the data were selected as the test and the training datasets, respectively. The input variables were fuzzified with Genfis3 code in MATLAB and then a fuzzy rule base was established using the Fuzzy C-Means (FCM) clustering approach. The membership functions of the input variable were created. The resulting fuzzy rules in the rule base were used to estimate GFR values. The membership functions were Gaussian. The fuzzy rules in the rule base build a fuzzy inference system and after training, which was converted to an ANFIS model. The trained ANFIS was used for predicting the GFR values at a 6-month interval and the predicting performance of ANFIS model for training dataset for the 6, 12, and 18-month future intervals was evaluated against the real measured GFR values.

## Results

### Implementing the MDSS

Regarding the framework and nature of neuro-fuzzy based DSS, whose functions are based on learning, the data of all patients in this study were divided into training and test datasets. The data from the test dataset were used to evaluate the predicting performance of accuracy of the predicting model.

Three criteria were selected to evaluate and compare the accuracy of the two neural network models: Mean Square Error (MSE), Mean Absolute Error (MAE), and Normalized MSE (NMSE). Of them, the NMSE is preferred since it provides the normalized error ranged from 0 to 100 percent. The formula for error criteria is expressed by Equations (1) to (3) as follows:

(1) $$MSE=\frac{\sum_{i=1}^{N}{\left(y_{i}-{\hat{y}}_{i}\right)}^{2}}{N}$$

(2) $$MAE=\frac{\sum_{i=1}^{N}\left|y_{i}-{\hat{y}}_{i}\right|}{N}$$

(3) $$NMSE=\frac{\sum_{i=1}^{N}{\left(y_{i}-{\hat{y}}_{i}\right)}^{2}}{\sum_{i=1}^{N}{\left(y_{i}\right)}^{2}}\times 100$$

**Figure 3:**
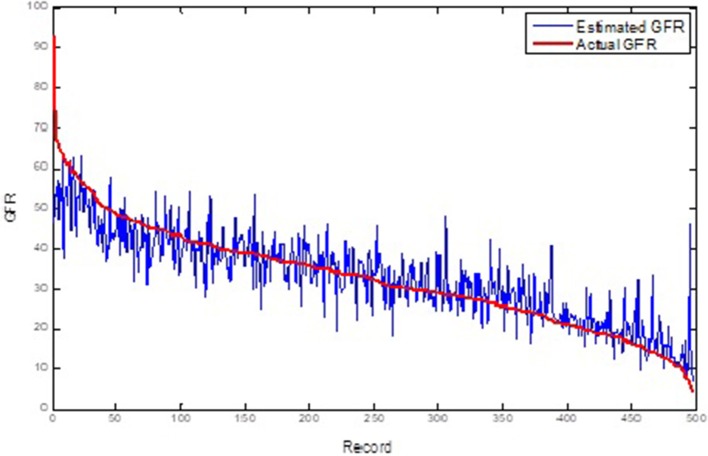
Comparison of the ANFIS prediction and real GFR(*t*+1) values for the training dataset.

**Figure 4:**
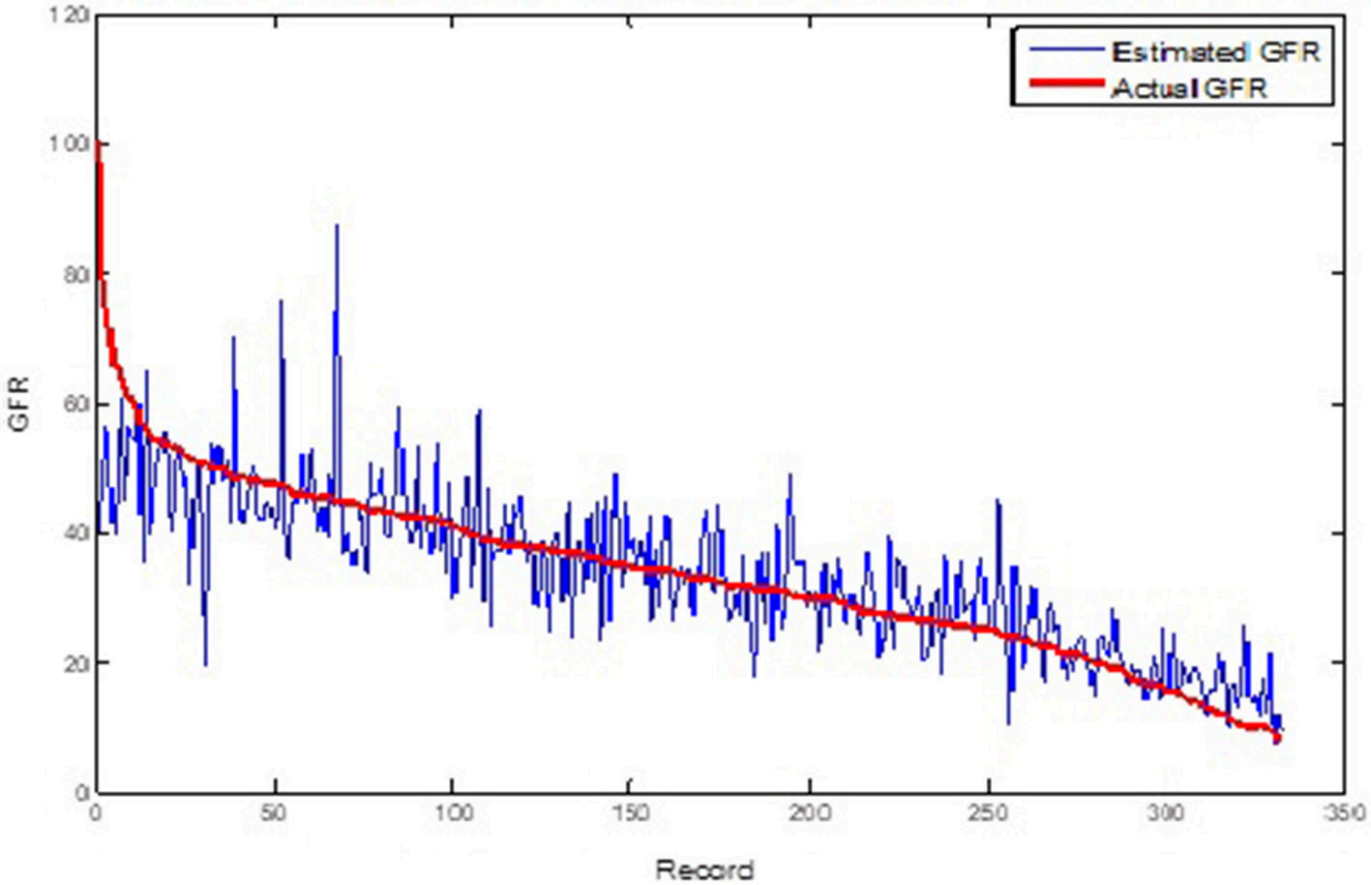
Comparison of the ANFIS prediction and real GFR(*t*+1) values for the test dataset.

Table 1 shows the ANFIS predictions of GFR values at sequential GFR_(t+1, 2, 3)_ for the training and test datasets based on the error criteria. The results show that the ANFIS neural network can accurately predict the GFR values for both training and test datasets at all three periods of 6, 12, and 18 months (Figures 3, 4). Despite increasing the predicting interval to 12 and 18 months, the ANFIS was still able to accurately predict the GFR values. Given the low error rate of the test data, the proposed ANFIS could be generalized to predict GFR values in new patients.

**Table 1:** Error criteria for the training/test datasets for 6, 12, and 18-month periods i.e., GFR _(t+1, 2, and3)_.

		**Training dataset**	**Test dataset**
6-month	MSE	58.6253	58.6253
	MAE	4.7654	4.7654
	NMSE	4.7676%	4.7676%
12-month	MSE	54.885	54.885
	MAE	5.5010	5.5010
	NMSE	4.3019%	4.3019%
18-month	MSE	64.0022	64.0022
	MAE	5.9302	5.9302
	NMSE	4.8787%	4.8787%

*MSE, Mean Square Error; MAE, Mean Absolute Error; NMSE, Normalized MSE*.

### Assessment of the MDSS Performance

**Figure 5:**
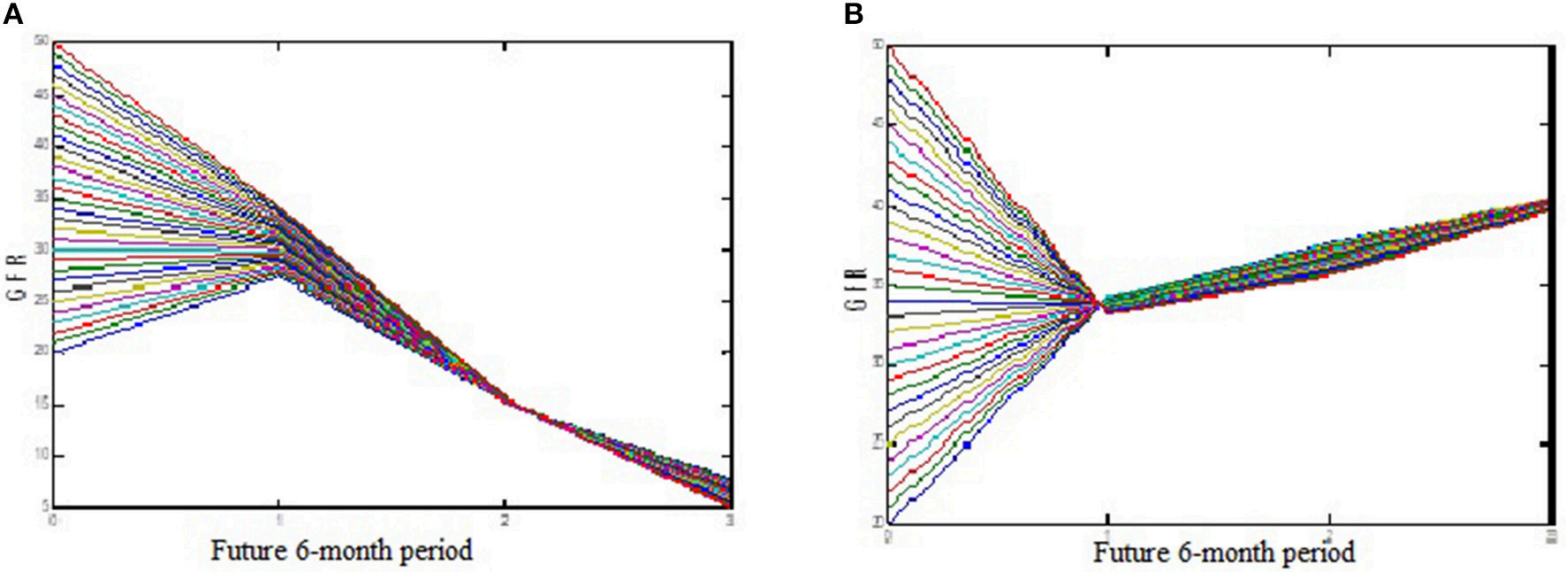
Predicted GFR variation among CKD patients with underlying disease of diabetes mellitus + urologic disease for sequential three 6-month interval. **(A)** Male patient; **(B)** female parient.

In addition to GFR prediction, the model produces a fuzzy rule base. The fuzzy rule base converts the complex relationships between experimental inputs and GFR as simple linear models in different modeling environments. The transparency of the GFR membership function in the ANFIS is the advantage of ANFIS compared to other predicting models such as linear regression and multilayer perceptron neural network. The trained ANFIS system could effectively predict the GFR values at sequential 6-, 12-, and 18-month intervals. In assessing the performance of the model, we evaluated the modeling results for different patients through assessing the effects of underlying disease, gender, and initial GFR values. The predicting results showed a different disease progression between male and female CKD patients when the underlying disease was diabetes mellitus + urologic disease (Figure 5). The predicted GFR values for male patients reached 7 during the 18-month interval, GFR_(t+3)_, indicating a failure of renal function (Figure 5A). However, female CKD patients showed a GFR_(t+3)_ value of about 37, indicating the improvement of kidney function (Figure 5B). The predicted data followed the real measurements well.

**Figure 6:**
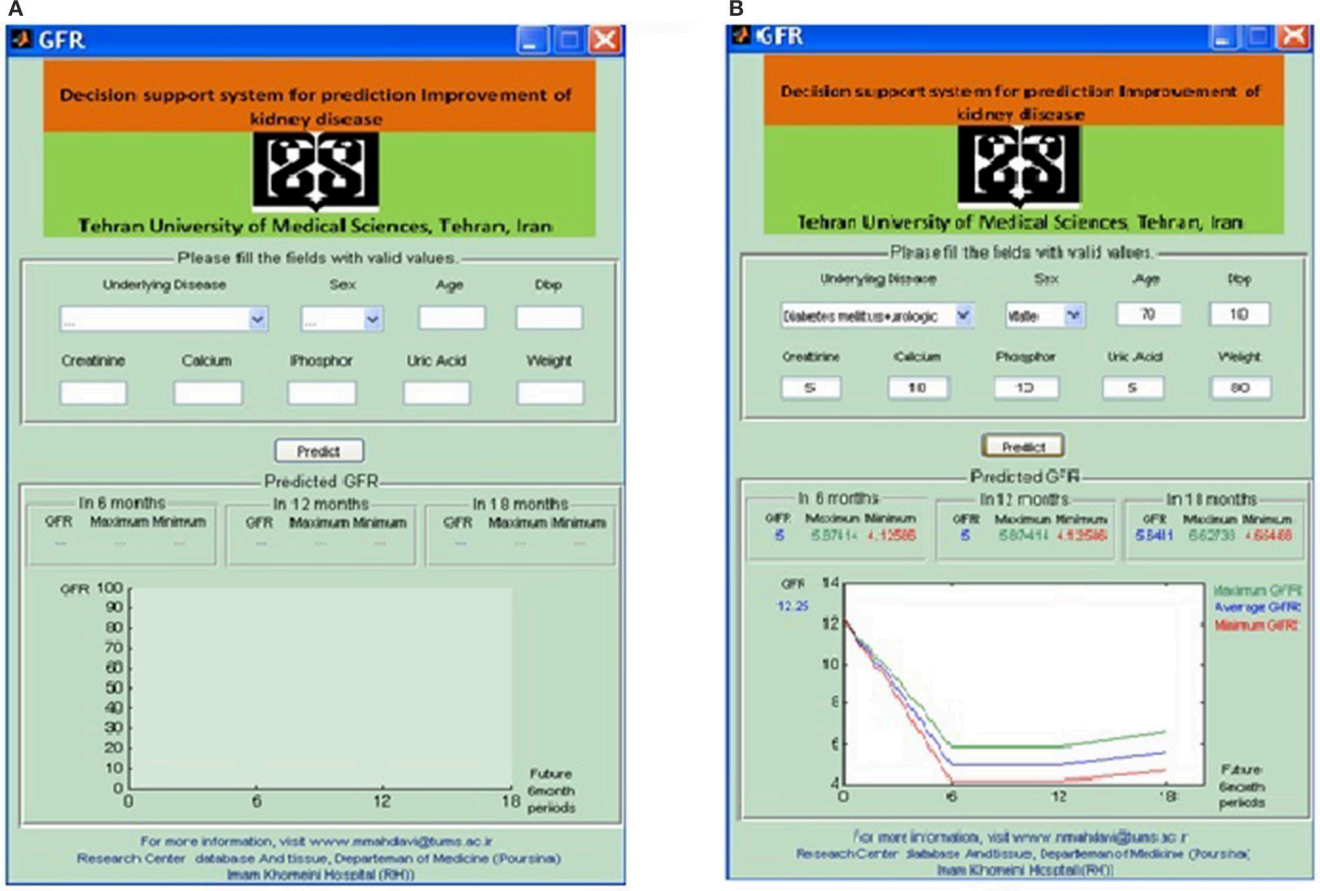
**(A)** Graphical user interface of the proposed MDSS based in ANFIS predicting model. **(B)** The output of GUI in a test patient.

Following the performance assessment of the predicting model, we designed a friendly-use graphical user interface (Meguid El Nahas and Bello, 2005) in MATLAB to build an MDSS for predicting the GFR values of CKD patients (Figures 6A,B). The MDSS uses the ANFIS model as its core computing model with appropriate topological structure. This system can be used by personnel with no programming or computer knowledge. The user defines the input variables, which are the results of laboratory and clinical assessments, and the system predicts the GFR values at three sequential 6-month intervals. The error of each computation is also represented by the model that helps the physicians to take appropriate decisions on the treatment options for each CKD patient. The model gives the minimum and maximum range of GFR at each interval. The average predicting error of the model, obtained through different comparisons with the real test data, was lower than 5%. The error range of our proposed model is sufficiently low to effectively support medical decisions in CKD management.

## Discussion

The present study introduces a new MDSS for monitoring and predicting renal failure progression in CKD patients. We evaluated the performance of three predicting models: improved linear regression model, MLP, and ANFIS neural networks. Of these, the latter showed the highest accuracy. The user-friendly MDSS was then built based on the predicting model. The MDSS showed high efficiency in predicting the GFR values. This DSS can reduce the cost of CKD management as well as reducing the mortality rate of the disease. In combination with the experience and knowledge of expert nephrologists, the proposed MDSS can significantly improve the quality of life of CKD patients. It is possible to use this system more in practice to help the management team to support patients effectively.

Several authors have attempted to predict the survival time of hemodialysis patients. Among them, Ma (Ma, 2012) worked with artificial neural and neuro-fuzzy models while other authors used machine learning based methods (Brier et al., 2003; Gaspari et al., 2004; Sengur, 2008; Hussain et al., 2011). A comparison of the results mentioned above, alongside ours, allowed us to affirm that our predicting models provide a reliable method to foresee the timeframe of worsening renal function in CKD patients. The strong point of our proposed MDSS was its high reliability in the prediction of GFR. This reliability comes from incorporating sequential measurements of GFR in CKD patients for an extended period of follow up (mean: 37.6 months). However, our DSS needs further improvement to include all influential parameters on CKD progression as well as to improve its ability to predict GFR variations in shorter intervals.

## Ethics Statement

This study was carried out in accordance with the recommendations of Declaration of Helsinki, Ethics committee of Tehran University of Medical Sciences, Tehran, Iran with written informed consent from all subjects. All subjects gave written informed consent in accordance with the Declaration of Helsinki. The protocol was approved by the ethics committee of Tehran University of Medical Sciences, Tehran, Iran.

## Author Contributions

JN and SM conceptualized the study and provided the resources. JN, SM, and AY performed the methodology and curated the data. JN and AY contributed to the software. JN, SM, AY, ES-A, and FT-M did the validation, investigation and the visualization. JN, AY, ES-A, and FT-M executed the formal analysis, the writing of the manuscript and prepared the original draft. SM, AY, ES-A, and FT-M wrote, reviewed and edited the manuscript. SM provided supervision. JN were responsible for the project administration and the funding acquisition.

### Conflict of Interest Statement

The authors declare that the research was conducted in the absence of any relationships that could be construed as a potential conflict of interest.
